# Role of stem cells in cancer therapy and cancer stem cells: a review

**DOI:** 10.1186/1475-2867-7-9

**Published:** 2007-06-04

**Authors:** Jayesh Sagar, Boussad Chaib, Kevin Sales, Marc Winslet, Alexander Seifalian

**Affiliations:** 1 Academic Department of Surgery, Royal Free Hospital, London, UK; 2 University College of London, London, UK

## Abstract

For over 30 years, stem cells have been used in the replenishment of blood and immune systems damaged by the cancer cells or during treatment of cancer by chemotherapy or radiotherapy. Apart from their use in the immuno-reconstitution, the stem cells have been reported to contribute in the tissue regeneration and as delivery vehicles in the cancer treatments. The recent concept of 'cancer stem cells' has directed scientific communities towards a different wide new area of research field and possible potential future treatment modalities for the cancer. Aim of this review is primarily focus on the recent developments in the use of the stem cells in the cancer treatments, then to discuss the cancer stem cells, now considered as backbone in the development of the cancer; and their role in carcinogenesis and their implications in the development of possible new cancer treatment options in future.

## Background

Cancer is the most common cause of mortality and morbidity in U.K. Despite recent advances in the treatments of cancer, the clinical outcome is yet far away from expectation. Use of stem cells in immuno-modulation or reconstitution is one of the methods used for decades in cancer therapy. Stem cells have self-renewal capacity with highly replicative potential in multilineage differentiation capacity [1].

**Figure 1:**
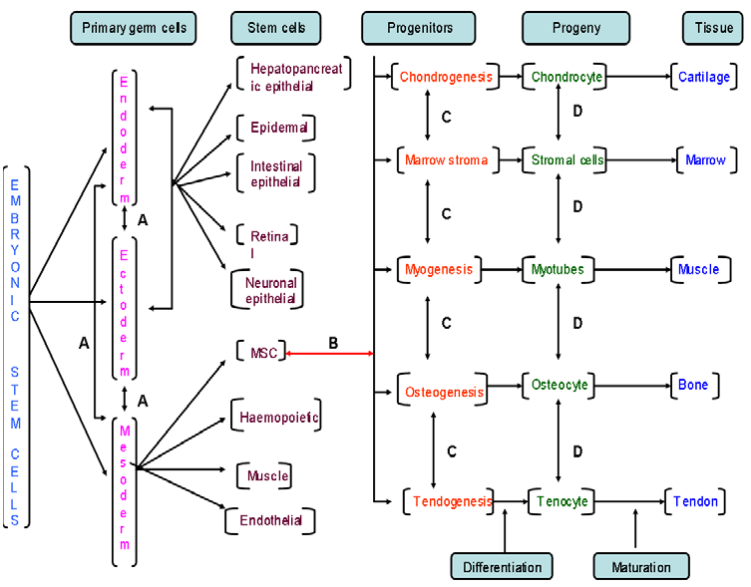
Hierarchy of stem cells with cell determination, differentiation and maturation. It also shows potential areas of **A**. Trans-germal plasticity – differentiation from one stem to other stem cell type; **B**. De-differentiation – regression of a fixed lineage cell type to a more primitive cell type; **C**. Trans-determination – differentiation from one progenitor cells to another; and **D**. Trans-differentiation – hypothetical differentiation of one cell type to another without dedifferentiation.

Stem cells can be divided into main three categories: embryonic, germinal, and somatic. Embryonic stem cells (ESCs) originate from the inner cell mass of the blastocyst. ESCs are omnipotent and have indefinite replicative life span, which is attributable to their telomerase expression[2]. Germinal stem cells are derived from primary germinal layers of embryo. They differentiate into progenitor cells to produce specific organ cells. Somatic/adult stem cells are progenitor cells as they are less totipotent i.e. less replicative life span than ESCs. They exist in mature tissues such as haematopoietic, neural, gastrointestinal and mesenchymal tissues. The most commonly used adult stem cells (ASCs) derived from bone marrow are haemopoietic stem cells (HSCs) and other primitive progenitor cells including mesenchymal stem cells (MSCs) and multipotent adult progenitor cells (MAPCs)[3,4] The microRNAs expression has been reported as a requisite to bypass G1/S checkpoint, thus for the self-renewal characteristic of stem cells[5]. Figure 1 shows hierarchy of stem cells with cell determination and differentiation. In this review, we highlight the potential of the adult stem cells in the cancer treatment and also focus on the new concept of the cancer stem cell.

## The choice of source of stem cells for cancer therapy

Ideally, ESCs would be the source of stem cells for therapeutic purposes due to higher totipotency and indefinite life span compared to ASCs with lower totipotency and restricted life span. However, use of ESCs have ethical constraints (Department of Health, UK, National Institutes of Health and International Society for Stem Cell Research) and their use for research and therapeutic purposes are restricted[6] and prohibited in many countries throughout the world. In addition, the stem cells with higher totipotency have been shown to be more tumorogenic in mice [7]. Thus, for ease of availability and lesser constrained on ethical issue, ASCs are the stem cells most commonly used for research and therapeutic purposes. The other reason for the use of ASCs is their easy accessibility compared to ESCs. According to literature, ASCs from bone marrow (HSCs & MSCs) are the most commonly studied[8] stem cells. MSCs support HSCs in the bone marrow and have the ability to differentiate both *in vivo *and *in vitro *into the different mesenchymal cells such as bone, cartilage, fat, muscle, tendon and marrow stroma[9].

## Stem cell sources

ESCs are derived from a 5-day old pre-implantation human embryos, however it posses potential risk of destroying the embryo. ASCs can be obtained from many tissues including bone, synovium, deciduous teeth, adipose tissue, brain, blood vessels, blood and umbilical cord blood[10-13]. Due to legal and ethical reasons, use of ESCs is restricted in research and clinical fields and ASCs remain the main supplement for the stem cells. Although ASCs can be obtained from the various sites, the ideal source of ASCs is yet to be found. Most commonly, ASCs are acquired from the bone marrow and peripheral blood. The bone marrow (BM) aspiration is one of the common procedures performed to obtain ASCs, but it is associated with morbidity in the form of wound infection and sepsis complications[14]. ASCs can also be obtained from adipose tissues such as abdominal fat and infra-patellar fat[15,16] which is less invasive and less morbid procedure than the bone marrow aspiration. It has been shown that there is no significant difference in the cell growth kinetics, cell senescence, gene transduction of adherent stromal cells and yield from stem cells obtained from bone marrow or adipose tissues[17]. The peripheral blood also provides a safe and easily accessible route for isolating ASCs with minimal morbidity. Use of ASCs through peripheral blood has shown to induce more T and NK (Natural Killer) cells compared to bone marrow ASCs[18]. Recently, the stem cells have been claimed to be obtained from the amniotic fluid without any harm to mother and embryo (posted on cnn.com on 08/01/2007).

## Stem cells in immuno-reconstitution

The stem cells have been used since many years in immuno-reconstitution following cancer development or following cancer treatments. The high dose chemotherapy have the adverse effects on the bone marrow causing myelosupression[19]. Usually this is followed by the blood cell recovery through the haematopoietic progenitor cells residing in the bone marrow by the complex interactions between the progenitor cells and the marrow microenvironment under the influence of various stimulatory and inhibitory factors [20-22]. However, time for haematopoietic recovery is proportional to the doses and number of cycles of chemotherapy [23] It has been shown that chemotherapy can induce inhibitory factors such as Tumour Growth Factor (TGF)-β, Interferon(IFN)-γ – IFN-α, Tumour Necrosis Factor(TNF)-α and Interleukin(IL)-4 with cytokines that causes myelosupression[24]. HSCs are the most commonly used and they are the stem cells of choice for the haematopoietic cell transplantation following high dose chemotherapy to restore bone marrow and immune system to pre-chemotherapy levels [25]. Randomised clinical trials regarding the use of HSCs for haematopoietic cell transplantation have been published with controversial results, however most of the trials suggested improved disease free survival rates, shorter hospital stay, overall survival rates and event free survival rates[26-31], while fewer of the studies have reported no statistically significant differences when assessed those parameters[32-35]. The adequate number of the stem cells therapy is also reported a crucial factors for speedy recovery[36]. Some of the chemotherapeutic agents, especially alkaylating agents, should be avoided as they are reported to adversely affect stem cell yield and haemotopoietic recovery[36,37]. The post-transplant period thrombocytopenia and neutropenia may be reduced by re-infusion of *ex vivo *expanded megacaryocyte progenitors[38] and re-infusion of *ex vivo *expanded peripheral blood stem cells (PBSC)[39] respectively.

## Choice of type of stem cell : bone marrow or peripheral blood

The source of stem cells is most commonly either from the bone marrow or the peripheral blood. The procedure of the bone marrow aspiration is invasive and is associated with the potential possible complications including fracture, wound infection and sepsis while the procedure for PBSCs isolation is much less invasive and less morbid. PBSCs have also been shown to induce higher number of CD4 T and NK cells compared to stem cells obtained from the bone marrow [18]. Thus, the stem cells from peripheral blood are considered the preferred source of stem cells however various clinical trials have publicized controversial conclusions comparing PBSCs and BM stem cells. It is also noticed that the occurrence of graft versus host reaction varies with PBSCs compared to BM stem cells [40,41]. Table 1 shows published human clinical trials comparing outcomes following transplantation of stem cells from BM and peripheral blood. These clinical trials provide different outcomes such as Storek et al suggested that PBSC yields higher lymphocyte subset counts[42] while Hernandez et al noticed no difference in the number of lymphocyte counts but noted faster reconstitution of cytotoxic subsets [43]. Similarly, these trials present controversial results including graft versus host disease, overall survival, disease free survival and immune recovery. Double stem cell transplantation has been documented to improve overall survival compared to single stem cell transplantation[44]. Granulocyte-colony stimulating factor (G-CSF) helps in proliferation and differentiation of haematopoietic progenitor cells [45]. G-CSF has also been reported to mobilise autologous peripheral blood stem cells and to preserve and increase the length of telomerase [45]. There are various different agents which are shown to enhance the G-CSG activity in mobilising stem cell. These are paclitaxel and docetaxel[46], recombinant human thrombopoietin[47], lithium[48] and recombinant methionyl human stem cell factor (r-metHuSCF)[49].

**Table 1:** Published human clinical trials comparing outcomes following isolation of stem cells from bone marrow vs peripheral blood.

**Authors**	**Type of cancer**	**No. of pats**	**RCT/CT**	**Conclusion**
Storek et al [42]	Haematological malignancies	140	RCT	PBSC yields higher lymphocyte subset counts and is associated with fewer infections
Hernandez et al [43]	Haematological malignancies	12	RCT	No significant difference in T, B and NK lymphoid cells reconstitution but PBSC influence faster reconstitution of cytotoxic subsets (CD8+/HLADR+ and NK lymphoid cells)
Talmadge et al [18]	Intermediate and high grade non-Hodgkin's lymphoma	116	RCT	The CD4:CD8 and CD45RA:CD45RO ratios were higher in the PBSC group. Accelerated reconstitution of NK cell activity following PBSC compared to BM.
Oehler et al [115]	Chronic myeloid leukaemia	72	CT	No statistically significant difference in acute or chronic GVHD, OS and disease free survival.
Heldal et al [116]	Haematological malignancies	61	CT	Statistically significant enhanced graft versus leukemia effect in allo PBSC group
Couban *et al *[41]	Haematological malignancies	228	RCT	Faster haematological recovery and improved survival in PBSC but no difference in GVHD
Nucci et al [117]	Haematological malignancies	56	RCT	Shorter duration of neutropenia in PBSC group but higher incidence of extensive chronic GVHD
Powles et al [118]	Haematological malignancies	39	RCT	Faster haematopoietic and immune recovery in PBSC and no difference in GVHDand OS
Mahmoud et al [119]	Haematological malignancies	30	RCT	Faster haematopoietic reconstitution in PBSC group with no difference in GVHD

Keys: RCT – Randomised Control Trial, CT – Controlled Trial, PBSC – Peripheral Blood Stem Cells, NK – Natural Killer, CD8, CD4, CD45RA & CD45RO – Different types of T Cells, HLA – Human Leucocyte Antigen, GVHD – Graft Vs Host Disease, OS – Overall Survival, Pats – Patients, No – Number.

## Role of purging in the isolation of stem cells

The isolation of stem cells from the allogeneic donor is the most preferable method, however only 30% of candidates are eligible due to the lack of donors and age restrictions[50]. Stem cells from autologous source are easily available but they carry the risk of coexistence of normal haematopoietic progenitors with malignant counterparts and may lead to the relapse of cancer. In population of patients with breast cancer, PBSC transplantation has been related to a rapid and sustained haematopoietic engraftment and has shown to be less contaminated than bone marrow stem cells[51]. There was however no overall improvement in survival outcome[52].

The contamination of the retrieval of stem cells with tumour cells have been major problem which reported by many studies [51,53,54], however the effect on clinical cell therapy has been less problematic[55,56].

Purging procedures are used in an attempt to remove these contaminant cancer cells from stem cells. Table 2 shows published clinical trials with various in vitro and in vivo techniques to purge the stem cells such as use of monoclonal antibodies, continuous flow immunoadsorption technique, dielectroforetic-field-flow-fractionation, use of rituximab, pulsed electric field, and hyperthermia. Amifostine has been shown to protect normal haematopoietic progenitor cells from damage by alkylating agents used for purging of stem cells[57,58]. The double procedure using 'positive CD34' and 'negative CD19' double selection method for purging is reported to be better than single procedure in the poor prognosis lymphoproliferative disorders, but it is associated with increased risk of life-threatening infections [59].

**Table 2:** Published clinical trials with various in vitro and in vivo stem cell purging techniques.

**Authors**	**In Vivo/In Vitro**	**Type of cancer(cells)**	**Purging technique**	**Conclusion**
Barbui et al [120]	IV & IT	Multiple myeloma	Two step negative selection procedure with combination of monoclonal antibodies	Safe procedure of purging stem cells. Higher event free survival rate
Stewart et al [121]	IT	Multiple myeloma	CEPRATE SC System – continuous flow immunoadsorption technique	No advantage of purging of stem cells
Vescio et al. [122]	IT	Multiple myeloma	CEPRATE SC System – continuous flow immunoadsorption technique	Significantly reduce tumour cell contamination and provides safe and rapid haematological recovery
Shpall et al [123]	IV	Breast cancer	WR-2721 (amifostine) to 4-hydroperoxycyclophosphamide (4-HC)	Reduced time to engraftment
Huang et al [124]	IT	Breast cancer	Dielectrophoretic field-flow-fractionation (DEP-FFF)	Efficient separation was observed in 12 minutes with purity of > 99.2%
Borbolla-Escoboza et al [125]	IT	B cell lymphoma	Rituximab	Rituximab can be used in stem cell purging
Craiu et al [126].	IT	Multiple myeloma	Pulsed electric fields	Promising technology for rapid stem cell purging
Wierenga et al [127]	IT	Acute myeloid leukaemia	Hyperthermia	Promising method for stem cell purging

Keys: IT – in vitro, IV – in vivo.

## Stem cells in tissue regeneration and as delivery vehicles

Apart from long lasting replicative property of stem cells, stem cells from haemopoietic tissues seem to have 'extraordinary' abilities to generate or switch between haematopoietic and non-haematopoietic lineages, exhibiting an unexpected degree of developmental or differentiation potential. On theoretical grounds, this allows HSC to be used to regenerate any non-haematopoietic tissue [60]. This technique has particular implications in bone tumours as reconstruction of bone following chemotherapy and surgery is always a major problem. The stromal stem cells derived from bone marrow have been used in the cell-based bone reconstruction following chemotherapy and surgery in osteosarcoma and Ewing sarcoma[61]. Jager et al have shown the regeneration of osteoblasts from the survived mesenchymal progenitor cells following COSS-96 (the cooperative osteosarcoma study) polychemotherapy *in vitro *and its potential *in vivo *use [62]. There are clinical trials showing role of the stem cells in the regeneration of myocardial tissue following myocardial infarction [63-65].

Systematic delivery of drug or gene therapy has promising future but is currently limited by various factors such as immune detection, non-specific accumulation in normal tissues and poor permeation. The effects of many anticancer agents are limited due to either their toxicities or their short half lives such as interferon β, which shows anti-proliferative and pro-apoptotic activities *in vitro*, but has shown restricted effects on human malignancies *in *vivo [66-68]. One proposed solution for these would be the cell-based carriers that may target the desired site.

The recent concept of use of stem cells as delivery vehicles came from the fact that the tumours, similar to the wounds, send out chemo-attractants such as the vascular endothelial growth factor (VEGF) to recruit MSC to form the supporting stroma of the tumour, and pericytes for angiogenesis. MSC transduced with an adenoviral expression vector carrying interferon-β gene has been demonstrated to increase the production of interferon-β at the local site[69]. However this *in vivo *function of MSC depends partly on signals from the target tissue microenvironment, for example, the tissues such as skin would have high cell turn over where there would be more signals for MSC compared to connective tissues where the high cell turn over is apparent only during healing process[70]. Likewise, MSC engineered to release interferon-β has been reported to create high local interferon-β levels in the mice glioma[71]. The neural stem cells have been reported as the delivery vehicles for the gene therapy for CNS disorders[72]. Similarly, interest has been shown in the use of the endothelial progenitor cells as the delivery vehicles for gene therapy because of their attraction towards the site of angiogenesis rather than the quiescent vasculature[73]. It may be possible to deliver immune-activating cytokines and other secreted proteins to brain and breast tumours though the stem cells.

## Life span of ASC

The major limiting factor in the use of stem cells in clinical area is the life span of the stem cells. Theoretically, the embryonic stem cells are best from this perspective due to their indefinitive replicative life span attributed to their telomerase expression[2]. However, practically, use of the embryonic stem cells in clinical area is very much restricted. Most of ASC do not possess sufficient telomerase activity and thus cannot prevent loss of telomerase. At each division, the telomerase shortens and the replication slows down (aging) and at the end, cells cease to divide (crisis phase) [74]. Thus we may not be able to obtain enough adult stem cells to perform our clinical task. One proposed solution is the use of genetic manipulation to extend the replicative span of the stem cells through the introduction of genes involved in controlling the replicative lifespan. In humans, this can be achieved by overcoming the replicative senescence by using the ectopic expression of telomerase hTERT gene [75]. In recent years, many studies suggested that hTERT-expressing stem cells continue to proliferate longer and maintain their ability to differentiate[76,7,78,9] Similarly, hMSCs have been immortalized by transduction with HPV16 E6/E7 *in vitro *without any neoplastic changes[79]. If it becomes successful to imply this principle in clinical practice, quantitative amount of the stem cells may not be one of the prognostic factors in the outcome in future.

## Cancer stem cells

Why a tumour does not respond to treatment? Why tumours recur? Why cancer cells develop resistance to treatment? These and many other raised questions may be answered by the new concept of "Cancer Stem Cells"[80].

Cancer stem cells can be defined as cells in the tumour growth with a tumour initiating potential. Normal stem cells are characterised by three properties: 1 Capability of self-renewal; 2 Strict control on stem cell numbers; 3 Ability to divide and differentiate to generate all functional elements of that particular tissue [81]. Compared to normal stem cells, the cancer stem cells are believed to have no control on the cell numbers. Cancer stem cells form very small numbers in whole tumour growth and they are said to be responsible for the growth of the tumour cells.

It has been well-known that in order to induce a tumour in an animal model, hundreds of thousands of cancer cells need to be injected [82]. This has been explained to be due to limitations in the assay to support tumour growth, or due to tumour formation deficiency [1]. With the recent concept of the cancer stem cells, it may be explained that higher numbers of cancer cells are needed to maximize the probability of injecting cancer stem cells in animal model. At present, the shrinkage in the size of a tumour is considered as a response to the treatment. However, tumour often shrinks in response to the treatment only to recur again. This may be explained by cancer stem cells that the treatment targeting the cancer cells may not be able to target the cancer stem cells

A fundamental problem in the cancer is the identification of the cell type capable of sustaining the neoplastic growth. There is evidence that the majority of the cancers are clones and that the cancer cells represent the progeny of one cell, however it is not clear which cells possess the tumour-initiating cell (TIC) function (cancer stem cells) and how to recognise them [83]. Though the idea of cancer stem cells is considered as a new concept in science, it was thought almost 35 years back in 1971 when they were called as leukaemic stem cells [84]. A small subset of cancer cells capable of extensive proliferation in leukaemia and multiple myeloma were found and named as leukaemic stem cells (LSC) [84]. Two possibilities were proposed: either all leukaemia cells had a low probability of proliferation and therefore all leukaemia cells behave as LSC, or only a small subset was clonogenic. The later theory was favoured by Dick and colleagues who were able to separate the LSC as CD34^+^CD38^- ^from patients' samples [85]. Despite being small in numbers (0.2%), these were the only cells capable to transfer Acute Myeloid Leukaemia from patients to NOD-SCID (non-obese diabetic-severe combined immunodeficiency) mice.

Recently, the cancer stem cells were also shown in the solid tumours such as breast cancer and brain tumours [86,87]. The cancer stem cells have been shown to have not only self-renewal capability but also generating wide spectrum of progeny, like normal stem cells [88]. In paediatric brain tumours, including medulloblastomas and gliomas, a subset of cells, called neurospheres, have been shown to have self-renewal capability. In conditions to promote differentiation, these neuospheres gave rise to neurones and glia, in proportion that reflect the amount in the tumour [89].

## Origin of cancer stem cells

**Figure 2:**
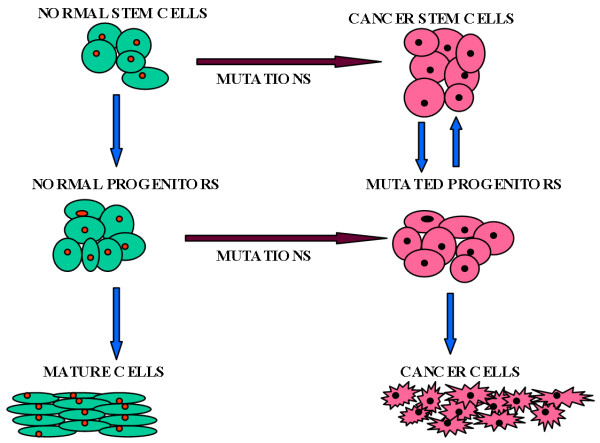
A simplified model of suggested hypothesis about origin of the cancer stem cells. The cancer stem cells may develop when self-renewing normal stem cells acquire mutations and are transformed by altering only proliferative pathways. It is also possible that the cancer stem cells originate by multiple oncogenic mutations in the restricted progenitor cells which acquire the capability of self-renewal (Created from NEJM [103]).

The cancer stem cells may be able to answer some of the questions related to a cancer growth, however origin of the cancer stem cells is yet to be defined. To recognise the origin of the cancer stem cells, two important factors need to be considered; 1 a number of mutations are required for a cell to be cancerous[90] and 2 a stem cell needs to overcome any genetic constraints on both self-renewal and proliferation capabilities[91]. It is unlikely that all the mutations could occur in the lifespan of a progenitor/mature cell. Therefore, cancer stem cells should be derived from either the self-renewing normal stem cells or from the progenitor cells that have acquired the ability of self-renewal due to mutations [92] (figure 2[92,93]).

The hypothesis that cancer stem cells are derived from normal stem cells rather than more committed progenitor cells have been addressed in the cases of AML where leukaemia initiating cells (LIC) from various subtypes of AML with different stages of differentiation have been shown to share the same cell-surface markers with normal haematopoietic stem cells [85,94]. However, some of the studies have suggested that cancer stem cells can be derived from the normal stem cells, as well as from the committed short-lived progenitors, giving rise to the tumours with comparable latencies, phenotypes and gene expression profiles[95-97] In the solid tumours, lack of the markers to characterise the tumour initiating cells (TIC) in the tumours has made it difficult to study the origins of the cancer stem cells, however there have been identification of cell-surface markers in the lung[4], brain[98-100] and prostate[101] which may allow the separation of the stem or progenitor cells with the tumour initiating function.

## Implications for cancer treatment

**Figure 3:**
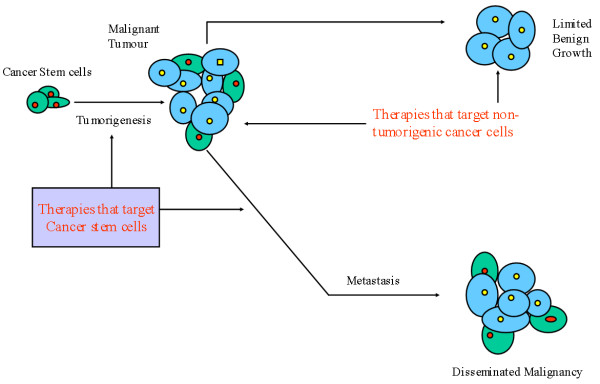
The conventional therapies may shrink the size of the tumour; by contrast, if the therapies are directed against the cancer stem cells, they are more effective in eradicating the tumour.

At present, the cancer treatment is targeted at its proliferation potential and its ability to metastasise, and hence the majority of treatments are targeted at rapidly dividing cells and at molecular targets that represent the bulk of the tumour. This may explain the failure of treatments to eradicate the disease or the recurrence of the cancer [1]. Although current treatments can shrink the size of the tumour, these effects are transient and usually do not improve patient's survival outcomes [102]. For tumours in which the cancer stem cells play role, three possibilities exist. First, the mutation of normal stem cells or progenitor cells into cancer stem cells can lead to the development of the primary tumour. Second, during chemotherapy, most of the primary tumour cells may be destroyed but if cancer stem cells are not eradicated, they become refractory cancer stem cells and may lead to recurrence of tumour. Third, the cancer stem cells may emigrate to distal sites from the primary tumour and cause metastasis[103]. Theoretically, identification of the cancer stem cells may allow the development of treatment modalities that target the cancer stem cells rather than rapidly dividing cells in the cancer. This may cure the cancer as the remaining cells in the cancer growth have limited proliferative capability (figure 3). If cytotoxic agents spare TICs, the disease is more likely to relapse. The TICs have been shown to have different sensitivity to different chemotherapeutic agents such as TICs in leukaemia are less sensitive to daunorubicin[104] and cytarabine[105].

Although the idea of the therapies focused on the cancer stem cells may look exciting, targeting the cancer stem cells may not be easy. The cancer stem cells are relatively quiescent compared to other cancer cells and do not appear to have the hyper-proliferation signals activated such as tyrosine kinase. These make the cancer stem cells resistant to the toxicity of the anti-cancer drugs, which traditionally target the rapidly dividing cells. In addition, the tumour suppressor gene *PTEN*[106], polycomb gene *Bmi1 *[107] and the signal transduction pathways such as the Sonic Hedgehog (Shh), Notch and Wnt that are crucial for normal stem cell regulation, have been shown to be deregulated in the process of cancinogenesis[87]. These deregulated signalling pathways and gene expressions may have impact on response to cancer therapy. One approach to target the cancer stem cells may be the identification of the markers that are specific for the cancer stem cells compared to normal stem cells such as haematopoietic stem cells express Thy-1 and c-kit whereas leukaemic stem cells express IL-3 (interleukin-3) receptor α-chain [108,109].

Much of the research is now focused on targeting the essential genes or pathways crucial for the cancer development through the cancer stem cells, with any possible therapies targeted against TICs. One such example is the use of Gleevec^® ^in chronic myeloid leukaemia that targets the ATP-binding domain of the Abl kinase. Most patients in this study experienced the complete cytogenetic responses[110,111]. although the therapy may not be curative due to reported presence of the fusion transcript[112]. A comparison of the pathways that regulate the stem cell homing with those responsible for metastasis may prove useful to minimise the toxic effects of the drugs. Treatment of mice with a Hedgehog (Hh) pathway inhibitor such as cyclopamine [113] inhibits the growth of medulloblastomas in mouse models, without any apparent toxicity. Thus, the Hh pathway may be inactive in most normal adult tissues, thus minimising the toxicity effects of these inhibitors [114]. Thus, the concept of the cancer stem cells has opened new areas of research in carcinogenesis and future treatment options.

## Conclusion and future prospectus

Presently, cancer therapy has entered in to an exciting new era, with traditional therapies such as chemotherapy, radiotherapy and surgery on one side while the stem cells on the other hand. Apart from their well-known role in immuno-reconstitution, the stem cells have attracted much attention especially with the new gene technologies such as the gene incorporation into the eukaryotic cells allowing more focused delivery of the anti-cancer agents. Now the cancer may be considered as a cancer stem cell disorder rather than that of rapidly growing cells. Although the origin of the cancer stem cells is yet to be defined, the concept of the cancer stem cells may allow new treatment options in the possible cure of the cancer. However, further research is required to identify and separate the cancer stem cells in various cancers from normal stem cells and other cancer cells. Further work is also required to differentiate the genes and signalling pathways in the process of the carcinogenesis from cancer stem cells for development of new therapies, with the eventual goal of eliminating the residual disease and recurrence.
